# A Comparison Study of Communication Skills Between General Surgery and General Practice Residents on First-time Patient Visits

**Published:** 2012-03-31

**Authors:** Ahmed Alansari

**Affiliations:** Ambrosiana University, Milan, Italy, and University of Calgary, Calgary, Alberta, Canada

## Abstract

**Background:**

There is little published research about differences in doctor-patient communication of different specialties. Accordingly, we compared doctor-patient communication skills in two different specialties, general surgery (GS) and general practice (GP).

**Methods:**

Twenty residents from the Bahrain Defence Force Hospital (10 men and 10 women; mean age 28 years; 10 GS and 10 GP) participated in 200 patient first visit consultations. The consultations were video-recorded and analysed by four trained observers using the MAAS Global scale.

**Results:**

1) Internal consistency reliability of the MAAS Global (> 0.91) and Ep^2^ = 0.84 for raters was high, 2) GP residents spent more time (12 minutes) than GS residents (7 minutes), in the visits, 3) There were several differences on the MAAS Global items between GP and GS residents (GS > GP, *p* < 0.05 on history taking, diagnosis and medical aspects; GP > GS, *p* < 0.05 on information giving), and 4) The present participants performed well compared to normative samples as well as to criterion-referenced cut-off scores. The general level of communication skills in both specialties, however, was ‘unsatisfactory’ and ‘doubtful’, as it is for normative samples.

**Conclusion:**

Excellent doctor-patient communication is essential but does not appear to receive the amount of attention that it deserves in practice settings. There are some differences between specialties as well as unsatisfactory communication skills for both specialties, since residents from both programs spent less time than recommended on each consultation. Our findings emphasize the need to improve the communication skills of physicians and surgeons in general.

## Introduction

Communication skills are essential to the effective practice of medicine as they may lead to improved patient outcomes and fewer complaints from patients regarding medical practice.^1^,^2^ Excellent communication, although difficult to describe, is related to the ability of doctors to identify the patient’s communication style and to try to use a concordant style in order to improve efficacy and satisfaction.^3^ To do this, doctors need to show respect, empathy, and understanding for their patients’ ideas, fears, expectations and opinions.^4^ All aspects of health care, including history taking, diagnosis, and provision of information to patients and their families depend on effective communication among other factors. As a reciprocal process, communication can only be effective if patients and practitioners have a shared understanding of the words and non-verbal cues that are used.^5^

Clear communication is essential for diagnostic accuracy, health outcomes, patient satisfaction,^6^ reduced complaints by patients,^7^ better adherence to treatment,^8^ reduction of patient stress, and overall physician clinical competency.^9^ As a result, health care organizations now stress that communication skills training should be an essential part of the curriculum of all medical schools and more medical education programs now require evidence of competency in communication for graduation and certification.^10^

Researchers are still exploring both what makes communication effective and the underlying mechanisms by which patients’ and providers’ outcomes are affected.^11^. Research on patterns of communication by general practitioners shows that patients strongly prefer a person-centred approach and prefer to be seen by a doctor who is able and willing to communicate well, to promote good health and to engage with them as healthcare partners. On the other hand, little research has been done with physicians from other specialties, and little is known about the differences and similarities between doctor-patient communication patterns between different specialties.^12^ There have been some attempts to study differences in communication skills between internal medicine and general practitioners for care outcomes and costs,^13^ but there has been no systematic research exploring any differences in communication skills between general surgery and general practice.

Some believe that general practitioners use a predominant patient-centred style and general surgeons tend to use a doctor-centred approach.^14^ This may suggest communication differences between doctors who deal with surgical procedures compared to doctors who do not. This, in part, may reflect the changing role of the doctor-patient relationship in the past two or so decades which now involves greater patient control, reduced physician dominance, and more mutual participation.^15^ Accordingly, the main purpose of the present study was to compare the communication patterns of doctor-patient consultations in two medical specialties, general surgery and general practice. To systematically study these differences, we conducted a comparative quantitative analysis of the similarities and differences in doctor-patient communication between residents in general surgery and general practice.

## Methods

### Participants

#### Residents

A total of twenty residents, ten from general practice (GP) and ten from general surgery (GS) from year 1, 2, and 3 of their residency program, were chosen at random and invited to participate in the study. The age of residents ranged from 25–33 years old with a mean age of 28 years. In general surgery there were 6 (60%) male doctors and 4 (40%) female doctors, whereas in general practice, there were 8 (80%) female doctors and 2 (20%) male doctors. The residents had completed a communication skills course during their medical education. None had attended extra courses in communication skills.

#### Patients

A total of 240 first visit patients to general surgery clinics and general practice clinics were selected and invited to participate. Patients’ age ranged from 15 to 90 years. There were 124 (62%) female patients and 76 (38%) male patients. Patient consent for the video-recording was obtained. The complexity of patients in clinical settings can range from easy to difficult. However the very easy cases and very difficult, complicated cases, which required specialist consultation, were not selected for the study. Cases of average difficulty were chosen. The level of difficulty was approved by two specialists in the relevant specialty.

### Design and Procedures

This comparative study used a quantitative analysis of checklist scores to compare doctor-patient consultations in general surgery and general practice. The study was conducted in the outpatient departments of the two specialties, general surgery and general practice, in the Bahrain Defence Force Hospital (BDFH). Patients come mainly from the military population and their relatives, and some from the civilian population.

All residents in both specialties received a written or verbal invitation, explaining the aim of the research, and they agreed to the video-recording of twelve first visit patient consultations. There was no drop-out of doctors from the study. Each resident was evaluated with twelve first visit patients. The first two patients in the evaluation were not used in the study as they were practice sessions to allow the doctors to become comfortable with the videotaping.

Twelve first visit patients were selected for residents to do an entire patient consultation in 15–20 minutes. A selection of ten patients is considered to be sufficient to allow comparisons of mean scores at the group level.^16^ Accordingly, 10 patient consultations for each of 20 residents (i.e., 200 in total) were analyzed. The consultations took place at the Bahrain Defence Force Hospital (BDFH). Residents were video-recorded, and the first two patients for each resident (i.e., 20 × 2 = 40) were not included in the study.

### Assessment of communication skills

The MAAS Global instrument for rating doctor communication skills was used. This instrument consists of a checklist and a 30-page scoring manual, listing criteria for each item.^17^ The MAAS Global instrument consists of three main aspects: 1) the communication skills for each separate phase such as introduction, follow up, consultation, and diagnosis, 2) general communication skills such as exploration, emotions, and empathy, and 3) medical aspects such as history taking, physical examination, and management. In the checklist seventeen case-independent items are used and rated on a 7-point scale: 0 = not present, 1 = poor, 2 = unsatisfactory, 3 = doubtful, 4 = satisfactory, 5 = good, 6 = excellent (see Appendix A).

The focus of the first thirteen items was on the communication skills, while the last 4 items related to the medical content. Since we limited our study to first-time patient visits, we excluded item number two which relates to follow up consultations. Several studies have provided evidence for the validity of the MAAS Global for assessing communication skills.^17^ In addition, the MAAS-Global has high internal consistency reliability (alpha > 0.90) and reproducibility (r > 0.80).^16^

Each rater reviewed 50 video-recorded consultations and gave a score for each consultation. The raters received standardized training, carried out by one trainer. The communication was evaluated by 4 trained observers who rated the videotapes using the MAAS Global checklist. The inter-observer reliability coefficient for each group of ratings was high (> 0.90).

### Ethics

The study was approved by the Ethics Committee of Bahrain Defence Force Hospital. Each doctor consented to take part and be video-recorded in the study. Consent for recording was also obtained from patients.

### Data Analysis

Descriptive statistics were computed on the data, between specialty differences were explored with one-way multivariate analysis of variance (MANOVA) of the scale items (independent variable = surgeon vs general; dependent variables = 16 items) and was followed-up with post-hoc one-way ANOVAs. The internal consistency reliability was computed with Cronbach’s α and overall generalizability analyses (Ep^2^) were conducted to determine the generalizability of various facets, including inter-rater reliability. A *p* value of < 0.05 was considered the critical value for significance.

## Results

Internal consistency reliability analyses for the total MAAS Global scale (k=16) produced α= 0.92 and the subscale α range from 0.65 to 0.87. A fully-crossed single-facet (4 raters × 10 consultations) generalizability analysis resulted in an Ep^2^ = 0.84. Accordingly, high reliability was achieved for both internal consistency and rater reliability. Although the time allocated for each first-time patient visit was 15–20 minutes, GS residents spent an average of 7 minutes with each patient and GP residents spent 12 minutes.

The between general surgery and general practice analyses were conducted with MANOVA (dependent variables = 16 items; independent variables = specialty) and are summarized in Table 1. There were overall differences between the two groups (Wilk’s lambda = 0.747; F = 3.87, *p* < 0.001) and subsequent post-hoc ANOVAs showed that there were several differences on single items and on one subscale. A close inspection of Table 1 reveals that GS residents outperformed GP residents on history taking (3.49 vs 3.18, *p* < 0.05) and diagnosis (3.34 vs 2.97, *p* < 0.05) but GP residents outperformed GS residents on information giving (3.24 versus 2.93, *p* < 0.05). The means and SD values are quite typical of studies of this sort.^16^

Table 2 contains the subscale scores and their descriptive statistics. When the items are summed into subscales or the total scales, there are no differences in the total MAAS Global scores between the two specialties. The lack of differences in the total scores between the two specialties is due to the cancelling effects of items 9, 13 and 15 in Table 1. Nor are there are differences in the communication skills for each separate phase and general communication skills. On medical aspects, however, general surgery outperformed general practice (3.25 versus 3.01, *p* < 0.05; this contains the 2 items where GS outperformed GP).

The communication skills of residents in both specialties are low as they ranged from 2 (unsatisfactory) to 3 (doubtful) in most of the communication items. The overall mean for the MAAS Global was 2.65 (unsatisfactory - doubtful) and as shown in Tables 1 and 2, residents from both specialties scored poorly on many items such as emotion, exploration, requests for help, and physical examination. Moreover, the lowest score on 14 of the 16 items was 0. These results are in concordance with normative data of the MAAS Global applied to GP consultations.

In one recent study, Reinders and colleagues^16^ found a mean MAAS Global score of 2.38 (SD = 0.95) of 74 video recordings of GP consultations. Similarly, other studies report mean scores of 2.36 (SD = 0.70) of consultations of 100 GPs^18^ and 2.35 (SD=0.65) of consultations of 88 GPs.^19^ The overall mean score for the MAAS Global for the residents in the present study was 2.70 (SD = 0.88), which compares favourably with the normative data of the MAAS Global. Using the borderline regression method, Hobma and colleagues^19^ set 2.5 as the ‘pass’ score for the assessment of doctor-patient communication in general practice. Based on this criterion-referenced cut-off score, approximately 60% of the residents in the present study ‘passed’ compared to a 38% pass rate in the normative sample of GPs.^19^

## Discussion

The results are 1) Internal consistency reliability of the MAAS Global and Ep^2^ for raters was high, 2) The patient encounters were brief, but GP residents spent more time than GS residents, 3) There were several differences on the MAAS Global items in performance between GP and GS residents, and 4) The present participants performed well compared to normative samples as well as to criterion-referenced cut-off scores.

The α reliability of the MAAS Global was high (> 0.90) in the present study as has been found in previous research.^17^ Additionally, we conducted the Ep^2^ analyses and found high consistency across raters indicating that our data has high reliability Reinders et al.^16^ found that 2 raters across 5 consultations are adequate to achieve Ep^2^ > 0.70. We exceeded this minimum with 4 raters for each of 10 consultations.

The present study is the first one to investigate the differences and similarities in doctor-patient communication skills in these two specialties, general surgery and general practice. Our findings indicate that doctor-patient communication differs between the specialties in history taking, information giving, and diagnosis as well as medical aspects. Moreover, the actual time spent with patients differed between specialties (GP > GS). As well, GP residents did better than GS residents in information giving, but the reverse was true for history taking, diagnosis and medical aspects as medical content. These differences probably reflect the differences in practice specialties, with GS more focused on the surgical aspects of the consultation whereas the GP residents may focus more on information sharing or giving. These differences are small (effect sizes ≈ 0.30), however, and there are no differences between the specialties on 13 of the 16 items or on the total scale score of the MAAS Global. Accordingly, GS and GP residents appear to be quite similar in their communication skills at least for first time patient encounters, although GP residents tend to spend more time with patients than GS residents.

We found that the general level of communication skills in both specialties received unsatisfactory or doubtful ratings on the MAAS Global. This finding is in concordance with several other studies of GP doctor-patient communications. Indeed, our GS and GP residents compared favourably with these normative samples on the MAAS Global. Based on the criterion-referenced cut-off scores, the present participants performed comparatively, with the majority passing the minimum standard. Nonetheless, both the GS and GP residents in the present study as well as the GPs in the normative studies require improvements in communication skills. This is likely true of many other physicians worldwide. Fortunately, there is evidence that structured individual communication improvement activities based on performance assessment are effective in improving communication skills in physicians. In a randomised controlled trial,^18^ the effect sizes for improvement of communication skills were moderate to large (Cohen’s d = 0.66). Some improvement may be gained by simply spending more time with the patient especially for surgeons, as in the present study the surgical residents spent only about 7 minutes with the patients.

Some communication skills such as information-giving and understanding the patient’s viewpoint are general competences that should be used by all physicians. These include giving greater importance to subjective aspects of the illness, chronic conditions, prevention, and screening^20^ in a patient-centred approach.

The present research was conducted in a military hospital in Bahrain, with military doctors, thus restricting the generalizability of the findings. Accordingly, further research in other settings and cultures should be conducted. Notwithstanding these limitations, the present results are in concordance with other work, particularly that conducted in the Netherlands.

## Conclusion

Excellent doctor-patient communication is essential in healthcare and does not appear to receive the amount of attention that it deserves in practice settings. We found that there are some differences between specialties as well as unsatisfactory communication skills for both specialties. Both GP and GS residents also spent less time than recommended on each consultation. Our findings emphasize the need to improve the communication skills of physicians and surgeons in general.

## Figures and Tables

**Table 1:** Comparison on the MAAS Global Items between General Surgery and General Practice Residents

MAAS Global Dependent Variable (range)±	Resident	Mean	SDŦ	95% CI¶	

				Lower Bound	Upper Bound
**SECTION 1: COMMUNICATION SKILLS FOR EACH SEPARATE PHASE**					

1. Introduction	General surgery	3.49	0.86	3.31	3.67
Min = 0, Max = 5	*General practice*	*3.32*	*0.92*	*3.14*	*3.50*
2. Request for help	General surgery	1.63	1.26	1.37	1.89
Min = 0, Max = 5	*General practice*	*1.31*	*1.35*	*1.05*	*1.57*
3. Physical exam (tells)	General surgery	1.75	1.20	1.47	2.03
Min = 0, Max = 5	*General practice*	*2.03*	*1.57*	*1.75*	*2.31*
4. Diagnosis	General surgery	3.05	1.25	2.81	3.29
Min = 0, Max = 6	*General practice*	*2.78*	*1.23*	*2.54*	*3.02*
5. Management (tells)	General surgery	3.29	1.21	3.06	3.52
Min = 0, Max = 6	*General practice*	*3.49*	*1.14*	*3.26*	*3.72*
6. Eval of consultation	General surgery	3.10	1.14	2.88	3.32
Min = 0, Max = 6	*General practice*	*3.24*	*1.11*	*3.02*	*3.46*

**SECTION 2: GENERAL COMMUNICATION SKILLS**					

7. Exploration	General surgery	1.80	1.33	1.52	2.08
Min = 0, Max = 5	*General practice*	*1.48*	*1.51*	*1.20*	*1.76*
8. Emotions	General surgery	1.35	1.28	1.09	1.61
Min = 0, Max = 5	*General practice*	*1.29*	*1.37*	*1.03*	*1.55*
9. Information giving*	General surgery	2.93	1.10	2.73	3.13
Min = 0, Max = 6	*General practice*	*3.24*	*0.89*	*3.04*	*3.44*
10. Summarization	General surgery	2.17	1.31	1.90	2.44
Min = 0, Max = 5	*General practice*	*2.35*	*1.40*	*2.08*	*2.62*
11. Structuring	General surgery	3.12	1.14	2.87	3.37
Min = 0, Max = 6	*General practice*	*3.02*	*1.40*	*2.77*	*3.27*
12. Empathy	General surgery	2.20	1.33	1.93	2.47
Min = 0, Max = 5	*General practice*	*2.47*	*1.38*	*2.20*	*2.74*

**SECTION 3: MEDICAL ASPECTS**					

13. History taking*	General surgery	3.49	1.05	3.30	3.68
Min = 1, Max = 6	*General practice*	*3.18*	*0.88*	*2.99*	*3.37*
14. Physical exam	General surgery	2.55	1.68	2.22	2.88
Min= 0, Max = 5	*General practice*	*2.26*	*1.67*	*1.93*	*2.59*
15. Diagnosis*	General surgery	3.34	0.99	3.12	3.56
Min = 0, Max = 6	*General practice*	*2.97*	*1.24*	*2.75*	*3.19*
16. Management (does)	General surgery	3.63	0.87	3.44	3.82
Min = 1, Max = 6	*General practice*	*3.62*	*1.04*	*3.43*	*3.81*

± 0 = not present; 1 = poor; 2 = unsatisfactory; 3 = doubtful; 4 = satisfactory; 5 = good; 6 = excellent;

Ŧ SD = standard deviation;

¶ 95% confidence intervals;

* *p* < 0.05

**Table 2:** Comparison on the MAAS Global Total and Subscale Scores between Surgery and General Practice Residents

MAAS Global Subscale and Total Scores	Resident	Mean	SDŦ	95% CI¶	

				Lower Bound	Upper Bound
Communication skills for each separate phase (1–6) (α=0.78±)	General surgery	2.72	.77	2.55	2.88
	*General practice*	*2.69*	*0.88*	*2.53*	*2.86*
General communication skills (7–12) (α=0.87)	General surgery	2.26	0.95	2.06	2.46
	*General practice*	*2.31*	*1.08*	*2.11*	*2.51*
Medical aspects* (13–17) (α=0.65)	General surgery	3.25	0.79	3.09	3.42
	*General practice*	*3.01*	*0.90*	*2.84*	*3.17*
Total Score (1–16) (α=0.92)	General surgery	2.68	0.78	2.51	2.85
	*General practice*	*2.63*	*0.91*	*2.46*	*2.80*

± Alpha coefficient (internal consistency reliability);

Ŧ SD = standard deviation;

¶ 95% confidence intervals;

* *p* < 0.05

## Appendix A MAAS-Global Rating List for Consultation Skills of Doctors

Jacques van Thiel, Paul Ram, Jan van Dalen, Maastricht University, Netherlands, 2003

0 = not present, 1 = poor, 2 = unsatisfactory, 3 = doubtful, 4 = satisfactory, 5 = good, 6 = excellent, na = not applicable

The rating boxes are intended only as a reminder for the observer.

Circle the relevant rating for each item.

**Table:**

**SECTION 1: COMMUNICATION SKILLS FOR EACH SEPARATE PHASE**								
**1. INTRODUCTION** (Giving the patient room to tell his story; general orientation on the reason for visit; asking about other reasons for visit )	na	0	1	2	3	4	5	6
**2. FOLLOW-UP CONSULTATION** (Naming previous complaints, requests for help, and management plan; asking about adherence to management plan; asking about the course of the complaint)	na	0	1	2	3	4	5	6
**3. REQUEST FOR HELP** (naming requests for help, wishes or expectations; naming reasons that prompted the patient to come now; completing exploring request for help)		0	1	2	3	4	5	6
**4. PHYSICAL EXAMINATION** (instructions to the patient; explanation of what is being done; treating the patient with care and respect)	na	0	1	2	3	4	5	6
**5. DIAGNOSIS** (naming findings and diagnosis/hypothesis; naming causes or the relation between findings and diagnosis; naming prognosis or expected course; asking for patient’s response)		0	1	2	3	4	5	6
**6. MANAGEMENT** (shared decision-making, discussing alternatives, risks and benefits discussing feasibility and adherence determining who will do what and when asking for patient’s response)		0	1	2	3	4	5	6
**7. EVALUATION OF CONSULTATION** (general question responding to requests for help; perspective for the time being)		0	1	2	3	4	5	6
**SECTION 2: GENERAL COMMUNICATION SKILLS**								
**8. EXPLORATION** (exploring requests for help, wishes or expectations; exploring patient’s response to information given within patient’s frame of reference; responding to nonverbal behaviour and cues)		0	1	2	3	4	5	6
**9. EMOTIONS** (asking about/ exploring feelings; reflecting feelings (including nature and intensity) sufficiently throughout the entire consultation)		0	1	2	3	4	5	6
**10. INFORMATION GIVING** (announcing; categorizing in small quantities; concrete explanations, understandable language; asking whether the patient understands)		0	1	2	3	4	5	6
**11. SUMMARIZATIONS** (content is correct, complete concise, rephrased checking sufficiently throughout the entire consultation)		0	1	2	3	4	5	6
**12. STRUCTURING** (logical sequence of phases; balanced division of time announcing (history taking, examination, other phases)		0	1	2	3	4	5	6
**13. EMPATHY** (concerned, inviting and sincerely empathetic in intonation, gesture and eye contact; expressing empathy in brief verbal responses)		0	1	2	3	4	5	6
**SECTION 3: MEDICAL ASPECTS**								
Rate according to professional guidelines if they are available. Otherwise rate to the best of your ability.								
**14. HISTORY TAKING**		0	1	2	3	4	5	6
**15. PHYSICAL EXAMINATION**	na	0	1	2	3	4	5	6
**16. DIAGNOSIS**		0	1	2	3	4	5	6
**17. MANAGEMENT**		0	1	2	3	4	5	6
**OTHER FEEDBACK**								
